# The Training of Patients

**Published:** 1904-10

**Authors:** 


### THE TRAINING OF PATIENTS.

Many of the ills to which we fall victims could be avoided if we had the proper foresight. Especially is this true in medicine. Not many physicians, busy with their daily cares and responsibilities, look into the future and try to steer their patients in that straight and narrow path which leads to success and contentment. There are many problems assailing the profession, many knotty puzzles which are discussed in medical societies and medical papers which would dissipate into thin air if we trained our patients as we do ourselves.

This training can take many forms; it is an individual problem that each man must work out for himself, yet it bears the same general outline. The properly trained patient avoids running up too large a bill for him to pay conveniently or promptly; he never calls the physician out at night unless it is necessary; he does not become dissatisfied on slight cause and go elsewhere. He has the belief impressed upon his mind that his physician is a man, with a man's limitations and necessities.

Particularly is this true in the matter of fees. If the patient gets the impression that his physician can be paid at any time or not at all; that his bills are carelessly made out and carelessly attended to, the patient will become equally careless. It is no trouble to avoid these errors; it is no loss of dignity in keeping before the patient that his bills should be promptly paid, and the patient will think more of his physician if this is done. Particularly is this true in collecting accounts; it should be done as far as possible by the doctor himself. How often has the physician allowed accounts to run for months or years without a murmur or the wink of an eyelid, and then suddenly turned some lynx-eyed collector on his surprised patients, only to lose both accounts and patients. It may seem disagreeable at first, but after a plunge or two it is not found to be the fact. The successful practitioner as a rule collects his own accounts, leaving to his collector incorrigible cases.

Again, under this heading of the training of patients, fall such problems as are discussed by Mills in a recent paper in the *Journal of the American Medical Association:* Should a physician go out at night to treat a case in a family already owing him a large bill, without any prospect of payment?

Should he treat a case involuntarily given up by another physician, when, in his opinion, the other physician has been unjustly discharged?

Should he adopt any of the measures commonly used by business men for the collection of his bills?

Should he undertake to treat at home, amid unfavorable surroundings, cases which positively need hospital treatment in order to recover?

Should he regulate his charges according to the means of the family treated?

These problems fall entirely within the education of the patient. Educate the men and women of your practice and these puzzles will fade into relics of the dreamy past.

Again, there are only twenty-four hours in the day, and a physician's time is his capital and stock. When his practice grows he should develop a secretary who can take much of the work from his shoulders—an intelligent, well educated woman can shift cases, get their names, residences, prospective means, etc., and prepare their introduction to the physician. An assistant should take family history, personal history, examination of heart and kidneys, etc., so that the foundations of the cases are ready for the busy practitioner to build. For a man overrun with work to dig for data with each case is a silly waste of time and energy. He should, of course, search for data of facts not brought out, to complete his picture, but the routine groundwork should be prepared for him by his assistants. His work will be done better, his patients will be better satisfied, and he will go to bed at night with twice or three times the results ordinarily accomplished, with far less expenditure of nervous force.—*Medical Times.*

---

KOCH'S ideas in regard to the prevention of the spread of typhoid fever by stamping out each focus have been carried out, according to an exchange, on a large scale in Alsace-Lorraine and other western provinces. Five bacteriologic stations were organized at various points. The superintendents of these stations, with other specialists and physicians of the region, met at Berlin June 29, to ascertain what had been learned and accomplished in regard to the eradication of the foci of typhoid fever. The assemblage was unanimous in decreeing that persons exhibiting typhoid bacilli in the urine or stools, with or without other symptoms or in apparent perfect health, should be isolated, if convenient, and if not, at least their excreta should be disinfected, as also their bed and body linen. The assemblage included R. Koch, Jaffky, Kruber of Munich, and Forster of Strasburg.

